# Supplementary material for: Comparative full-length transcriptome analysis by Oxford Nanopore Technologies reveals genes involved in anthocyanin accumulation in storage roots of sweet potatoes (Ipomoea batatas L.)
Source: PeerJ. 2022 Jul 12;10:e13688. doi: 10.7717/peerj.13688 (PMC9285475; doi:10.7717/peerj.13688)
Supplement: Table S1 [file peerj-10-13688-s001.docx]

**Supplemental Table 1 The sample groups for transcriptome**

| [Experimental](javascript:;) [material](javascript:;) | Low anthocyanin content group | High anthocyanin content group |
| --- | --- | --- |
| Purple-fleshed sweet potato (PESP) | PL (40 days after planting) | PH (110 days after planting) |
| White-fleshed sweet potato (WESP) | WL (40 days after planting) | WH (110 days after planting) |
