# Supplementary figures and images for: Comparative full-length transcriptome analysis by Oxford Nanopore Technologies reveals genes involved in anthocyanin accumulation in storage roots of sweet potatoes (Ipomoea batatas L.)
Source: PeerJ. 2022 Jul 12;10:e13688. doi: 10.7717/peerj.13688 (PMC9285475; doi:10.7717/peerj.13688)

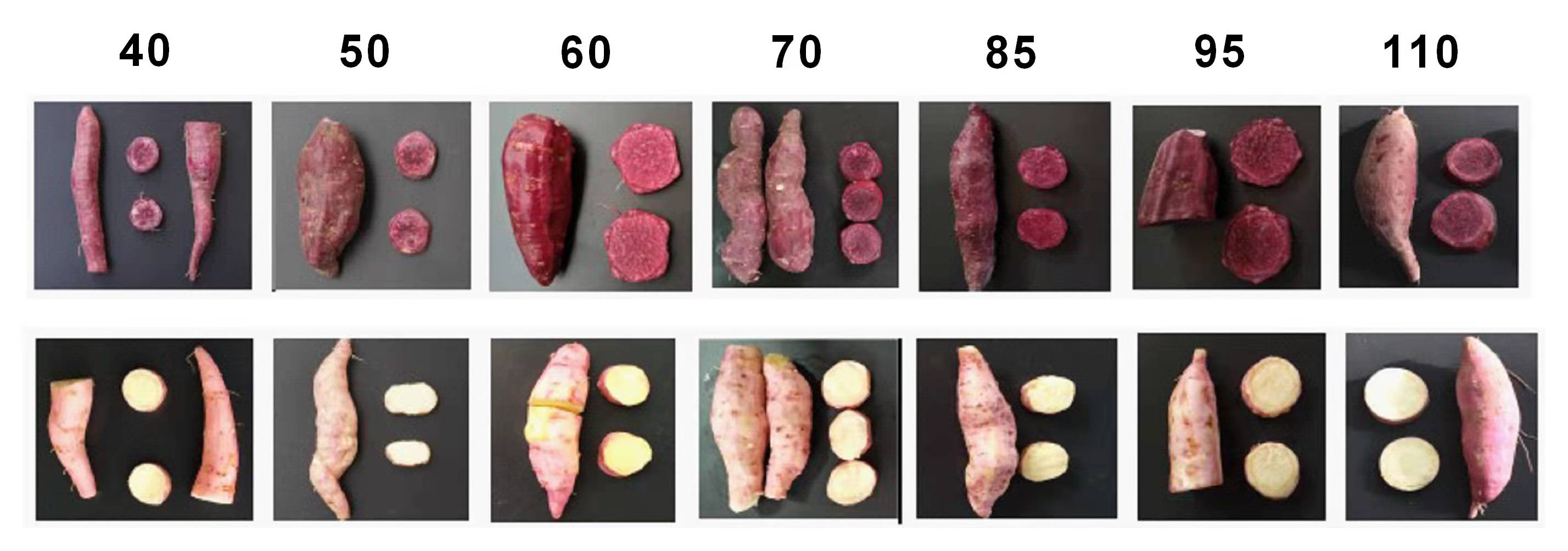

Supplement: Supplemental Information 2 [file peerj-10-13688-s009.jpg]

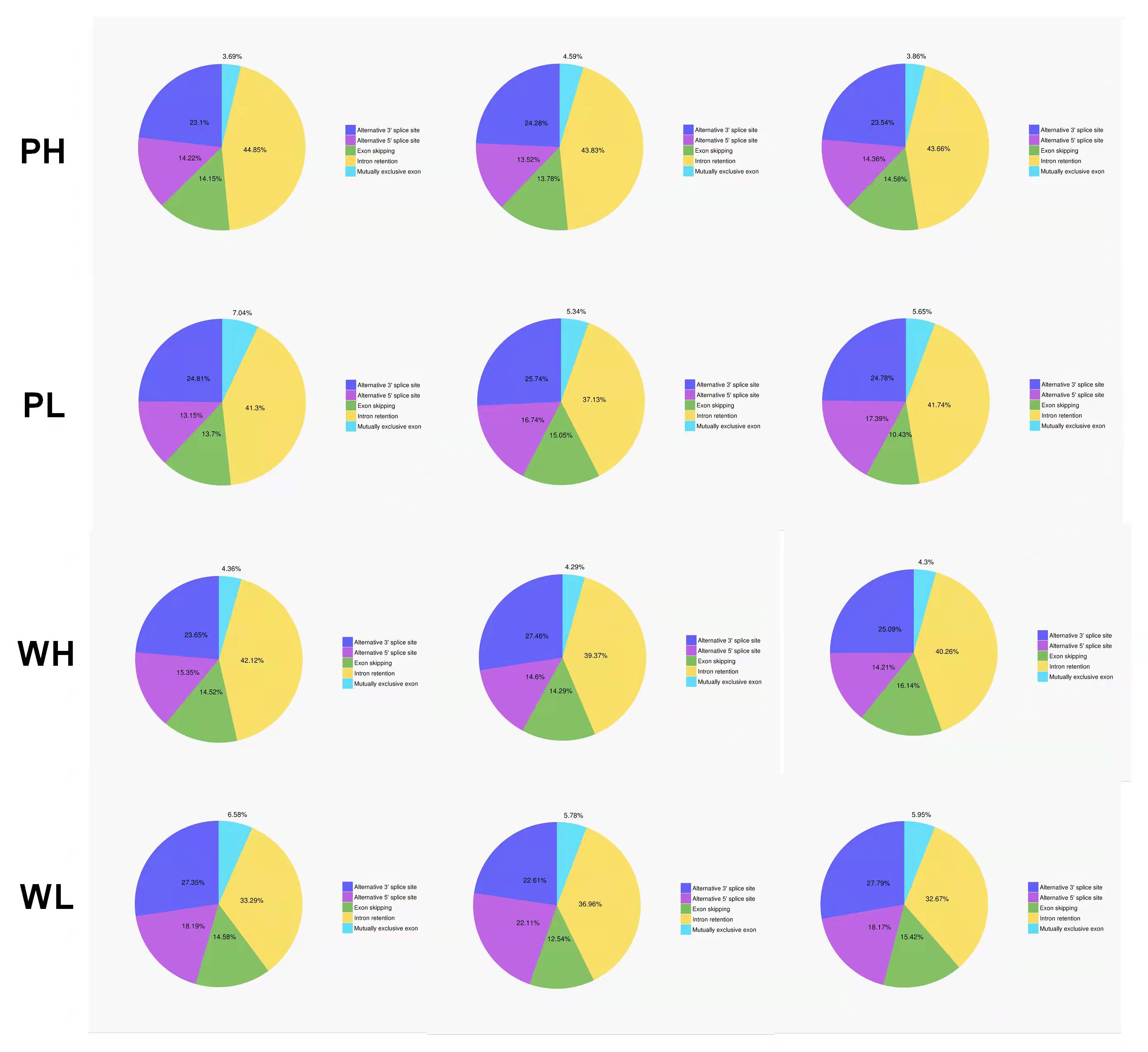

Supplement: Supplemental Information 3 [file peerj-10-13688-s010.jpg]

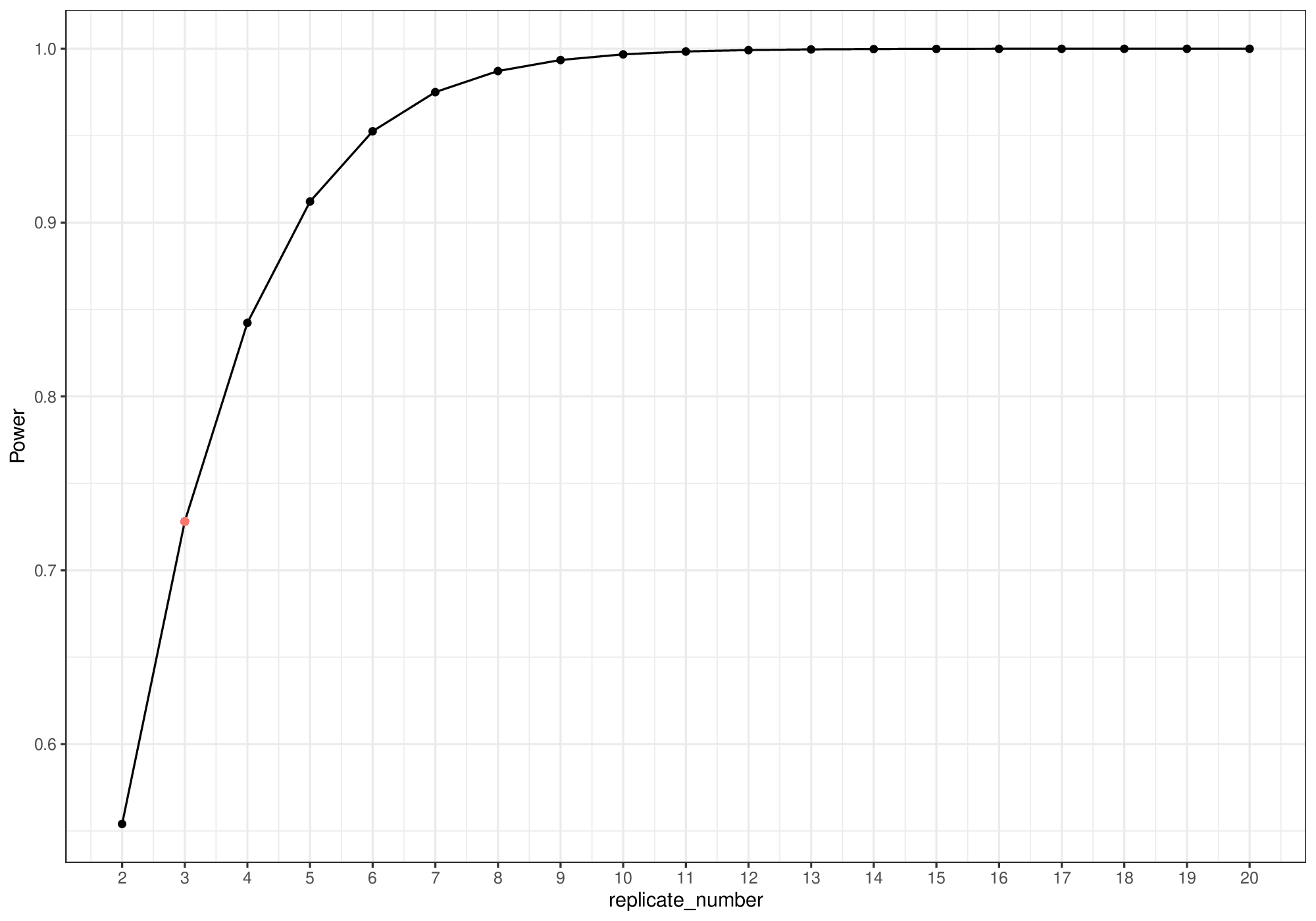

Supplement: Supplemental Information 4 [file peerj-10-13688-s011.zip › Power analysis/PL_vs_WL_power.png]

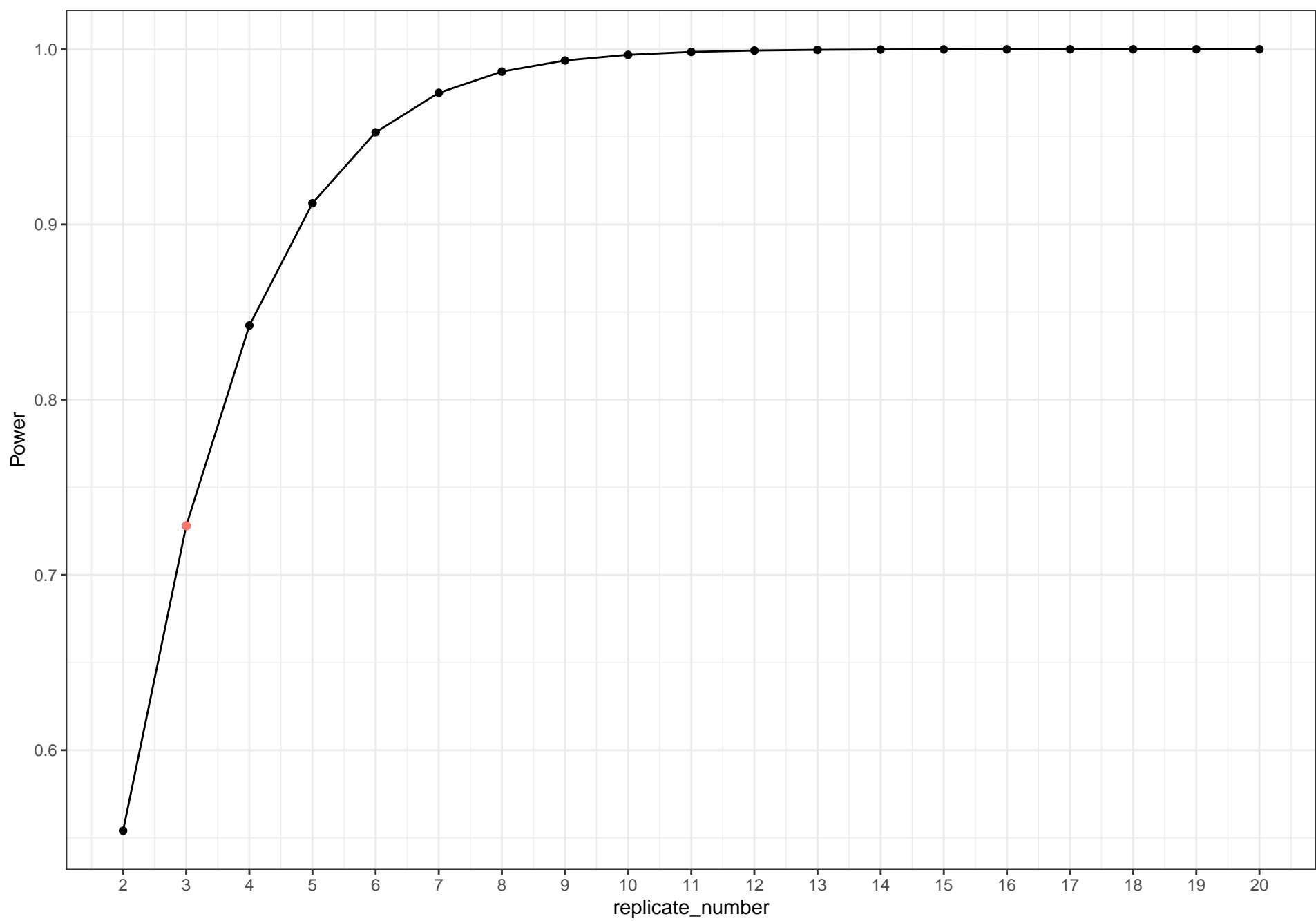

Supplement: Supplemental Information 4 [file peerj-10-13688-s011.zip › Power analysis/PL_vs_WL_power.pdf]

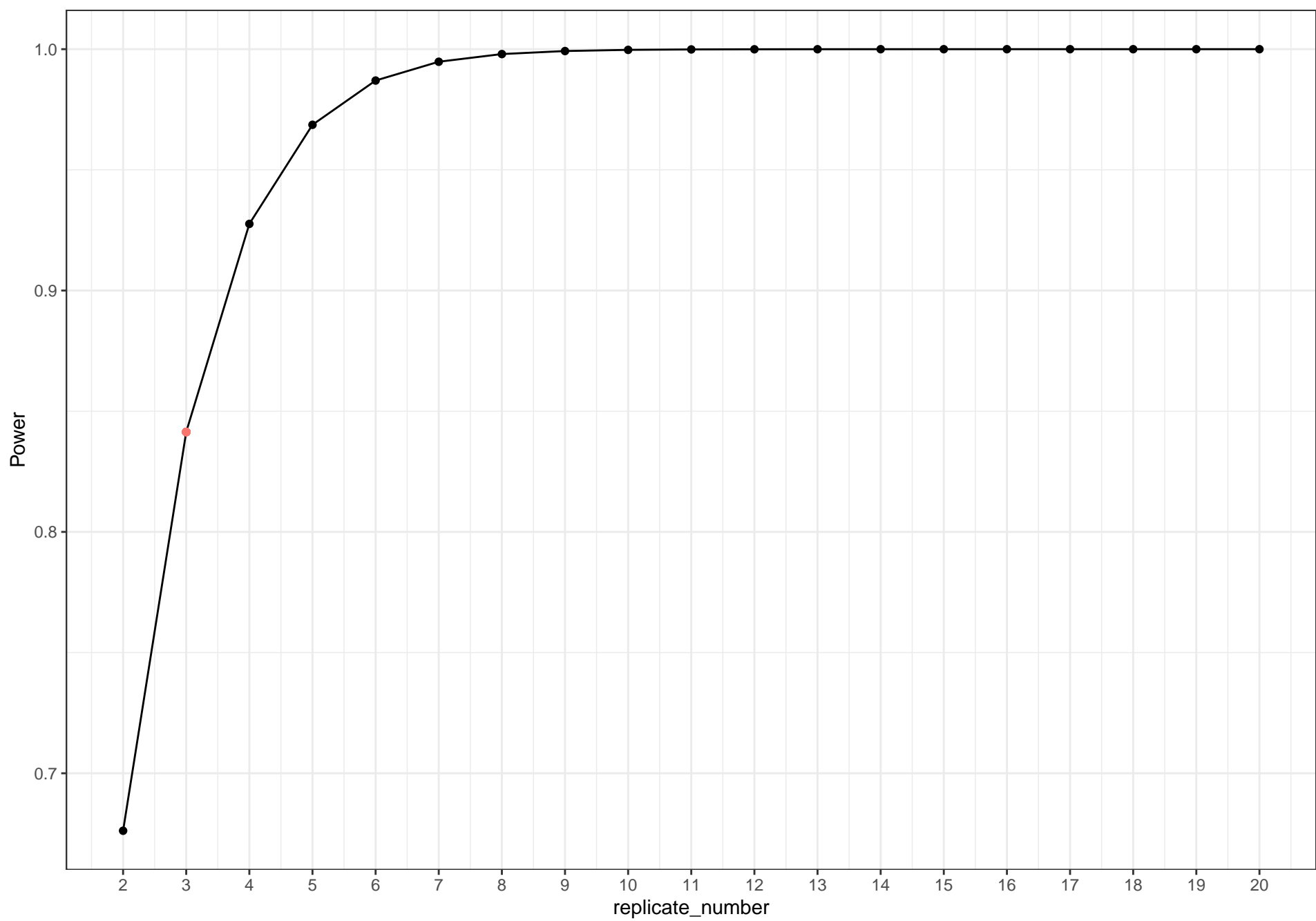

Supplement: Supplemental Information 4 [file peerj-10-13688-s011.zip › Power analysis/PH_vs_WH_power.pdf]

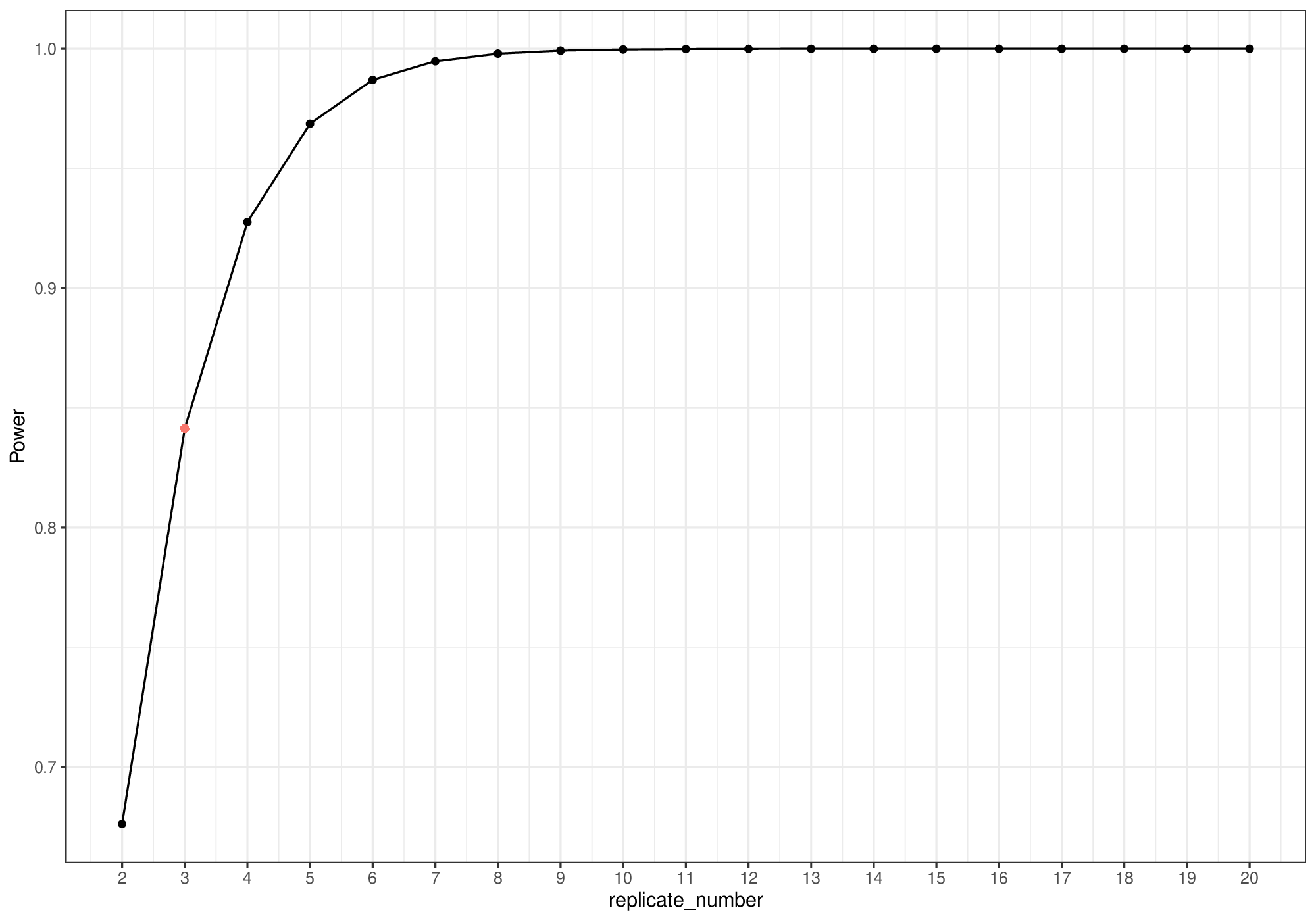

Supplement: Supplemental Information 4 [file peerj-10-13688-s011.zip › Power analysis/PH_vs_WH_power.png]
